# Peripartum-Associated Heart Failure Develops Independently of RHOT Proteins

**DOI:** 10.3390/ijms27114991

**Published:** 2026-05-30

**Authors:** Natali Froese, Eluiesa Sina, Paolo Galuppo, Christopher Werlein, Anna Gigina, Jan Hegermann, Robert Geffers, Tim Scholz, Jan C. Kamp, Lavinia Neubert, Johanna Schneider, Melanie Ricke-Hoch, Alexander Dietl, Johann Bauersachs, Christian Riehle

**Affiliations:** 1 Department of Cardiology and Angiology, Hannover Medical School, 30625 Hannover, Germany; 2 Institute of Pathology, Hannover Medical School, 30625 Hannover, Germany; 3 Research Core Unit Electron Microscopy, Institute of Functional and Applied Anatomy, Hannover Medical School, 30625 Hannover, Germany; 4 Research Group Genome Analytics, Helmholtz Center for Infection Research, 38124 Braunschweig, Germany; 5 Institute of Molecular and Cell Physiology, Hannover Medical School, 30625 Hannover, Germany; 6 Department of Respiratory Medicine and Infectious Diseases, Hannover Medical School, 30625 Hannover, Germany; 7 Biomedical Research in Endstage and Obstructive Lung Disease Hannover (BREATH), German Center for Lung Research (DZL), 30625 Hannover, Germany; 8 Department of Medicine IV, Medical Center—University of Freiburg, 79106 Freiburg, Germany; 9 Department of Internal Medicine II, University Hospital Regensburg, 93053 Regensburg, Germany

**Keywords:** heart failure, hypertrophy, mitochondria, mitochondrial motility, peripartum cardiomyopathy (PPCM)

## Abstract

Pregnancy-associated hemodynamic overload and hormonal changes induce hypertrophy and metabolic remodeling of the maternal heart. Mitochondrial motility, mediated by ras homolog family member T (RHOT) 1 and RHOT2, is essential for cardiac adaptation to increased workload, cardiomyocyte hypertrophy, and sarcomere maturation. To test the hypothesis that *Rhot1/2* expression is required for pregnancy- and postpartum-associated adaptations of the maternal heart, female mice with tamoxifen-inducible, cardiomyocyte-selective deletion of *Rhot1* and *Rhot2* (iRhot1/2-KO) were mated. Following gene deletion in adult mice, cardiac tissue and function were analyzed after three to five successive pregnancies and postpartum nursing periods. Age-matched nulliparous iRhot1/2-KO mice and age-matched mice expressing *Rhot1* and *Rhot2* served as controls. Motility of mitochondria isolated from iRhot1/2-KO hearts was impaired, as determined by the number of mobile mitochondria in an in vitro motor protein-driven single mitochondrion motility assay performed on surface-immobilized microtubules. Despite loss of *Rhot1/2* expression, contractile function assessed by transthoracic echocardiography, mRNA expression of peripartum-associated heart failure markers, cardiac structure, mitochondrial morphology, mitochondrial enzymatic activity, and mitochondrial DNA content were all comparable to controls expressing *Rhot1/2* at the investigated time points. RNA sequencing-based gene profiling identified a transcriptional program through which RHOT proteins preserve cardiac energetic and contraction gene expression during pregnancy and postpartum. Together, cardiomyocyte-selective loss of *Rhot1/2* expression in the adult heart does not cause peripartum-associated heart failure, despite reduced cardiac energetic and contraction gene expression.

## 1. Introduction

During pregnancy, the hemodynamic load on the maternal heart increases to accommodate the expanded blood volume and to support fetal growth [1]. Heart rate, stroke volume, cardiac output, and blood volume begin to increase during the second trimester, peak around mid-pregnancy, and remain elevated until delivery [1]. These profound physiological changes result in eccentric cardiac hypertrophy, with left ventricular (LV) mass increasing by up to 30% during pregnancy and typically returning to baseline approximately six months postpartum (PP) [2,3]. Failure of the maternal heart to adapt during pregnancy or PP can lead to peripartum cardiomyopathy (PPCM), a potentially life-threatening condition and a leading cause of maternal mortality worldwide [4,5,6,7]. PPCM is defined as maternal heart failure resulting from LV systolic dysfunction, characterized by an LV ejection fraction <45%, occurring toward the end of pregnancy, around delivery, or within the months following delivery, in the absence of any other identifiable cause [4,7,8,9,10]. The mechanisms contributing to PPCM include hormonal imbalance and remain a subject of intensive investigation [4]. Murine models continue to provide important insights into the pathogenesis of PPCM. Transgenic mice with deletion of *peroxisome proliferator activated receptor gamma coactivator 1 alpha* (*Ppargc1a*, PGC-1α), a master regulator of mitochondrial biogenesis and energetic gene expression, or deletion of *signal transducer and activator of transcription 3 (Stat3)* develop PPCM due to angiogenic imbalance [11,12,13,14]. Cardiomyocyte-selective *Stat3* deficiency increases oxidative stress and induces cleavage of the lactation hormone prolactin into a 16-kDa anti-angiogenic fragment, which mediates apoptosis and inhibits endothelial cell proliferation and migration [14]. The neuregulin/ErbB paracrine endothelium-controlled system is functionally activated during physiological ventricular remodeling in pregnancy, and expression of *Erb-b2 receptor tyrosine kinase 4 (Erbb4)* is reduced in *Stat3*-deficient hearts [14,15]. Of note, PGC-1α is also a key regulator of cardiomyocyte metabolic pathways [11]. Cardiomyocytes derived from PPCM patients exhibit impaired metabolic flexibility upon inhibition of lipid metabolism [16]. These findings suggest that metabolic imbalance and mitochondrial dysfunction may contribute to the development of PPCM [10].

The heart has a high energy demand. Given the limited energy storage of cardiac tissue and the complete turnover of cardiac ATP approximately every 10 s, ATP must be continuously generated by cardiomyocyte mitochondria [17]. Consequently, the heart has the highest mitochondrial content of all organs in mammals [18]. Ras homolog family member T (RHOT) 1 and RHOT2 are atypical rho-like small GTPases anchored to the outer mitochondrial membrane and are highly expressed in the heart [19,20]. RHOT proteins, together with trafficking kinesin proteins (TRAKs), link mitochondria to kinesin and dynein motors, enabling intracellular mitochondrial motility [19]. Our previous studies demonstrated a critical role for mitochondrial motility, mediated by RHOT proteins, in cardiomyocyte maturation. The identified mechanisms comprise binding of RHOT proteins to contractile muscle fiber proteins, which is essential for mitochondrial positioning, and locally confined ATP production during cardiomyocyte hypertrophy [21]. The present study tested the hypothesis that RHOT proteins are required for pregnancy- and PP-associated adaptations of the hypertrophied maternal heart with increased energy demand [2,3]. Transgenic mice with *Rhot1* germline deletion exhibit defective motor neurons, leading to respiratory failure and death shortly after birth [22]. Mice with *Rhot2* germline deletion survive until adulthood, whereas germline deletion of both *Rhot* isoforms results in prenatal death by embryonic day 8.5 due to developmental defects [23,24]. The contribution of RHOT proteins to cardiomyocyte hypertrophy is supported by studies in Burmese pythons, which identified *Rhot2* as a highly upregulated transcript during the early hypertrophic response following feeding [25,26]. Furthermore, *Rhot2* overexpression attenuates heart failure in mice subjected to pressure overload induced by transverse aortic constriction [27]. To elucidate the contribution of mitochondrial motility to pregnancy- and PP-associated adaptations of the maternal heart, we subjected mice with tamoxifen-inducible, cardiomyocyte-selective deletion of *Rhot1* and *Rhot2* (iRhot1/2-KO) to successive pregnancies and PP nursing periods. Hearts were examined post-delivery and at the end of the nursing period to determine the contribution of RHOT proteins to pregnancy- and PP-associated adaptations, which are characterized by temporal hemodynamic and hormonal changes [4,10]. Nulliparous (NP) iRhot1/2 KO and mice expressing *Rhot1* and *Rhot2* were used as controls.

## 2. Results

### 2.1. Rhot1/2-Mediated Mitochondrial Motility Is Dispensable for Cardiac Structure and Contractile Function During Pregnancy and PP

**Figure 1:**
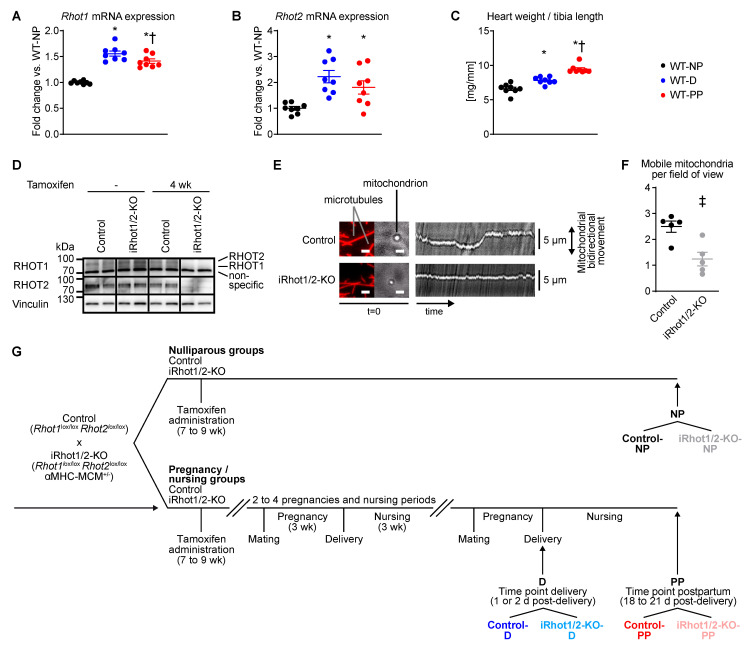
Experimental design and increased mRNA expression of *Rhot1* and *Rhot2* in hypertrophied maternal hearts at delivery and postpartum. (**A**,**B**) *Rhot1* and *Rhot2* mRNA expression normalized to *Hprt1* in left ventricular homogenates and (**C**) heart weights normalized to tibia length from wildtype mice (WT) at the time points indicated, each measured after the first pregnancy (*n* = 8). * *p* < 0.05 vs. WT-NP, † *p* < 0.05 vs. WT-D (one-way ANOVA, followed by Holm–Šídák post hoc analysis). (**D**) Representative immunoblots for RHOT1 and RHOT2 in left ventricular homogenates from iRhot1/2-KO and control mice. (**E**) Images and kymographs of mitochondria isolated from hearts 8 weeks post-tamoxifen administration. Images show surface-immobilized Cy5-labeled microtubules and mitochondria at time = 0 (scale bars: 2 µm). Kymographs represent mitochondrial motility along microtubules over a 45-s time period (length). (**F**) Number of mobile mitochondria per field of view isolated from hearts 8 weeks post-tamoxifen administration (Control: *n* = 115; iRhot1/2-KO: *n* = 68; data were obtained from *n* = 5 mice/genotype; ‡ *p* < 0.05 vs. Control). (**G**) Experimental design with tamoxifen administration for the induction of gene deletion and time course of mating and nursing. Mice were mated 2–3 weeks after the final tamoxifen injection. Data are reported as mean values ± SEM. Control-D, Control/delivery; Control-NP, Control/nulliparous; Control-PP, Control/postpartum; iRhot1/2-KO-D, iRhot1/2-KO/delivery; iRhot1/2-KO-NP, iRhot1/2-KO/nulliparous; iRhot1/2-KO-PP, iRhot1/2-KO/postpartum; WT-D, wildtype/delivery; WT-NP, wildtype/nulliparous; WT-PP, wildtype/postpartum.

*Rhot1* and *Rhot2* mRNA expression was increased in maternal wildtype (WT) hearts at delivery (WT-D) and PP (WT-PP) relative to NP mice (WT-NP; Figure 1A,B). Consistent with previous studies [28], heart weights normalized to tibia length were increased in WT-D compared with WT-NP, with a further increase in WT-PP (Figure 1C). To test the hypothesis that RHOT proteins and mitochondrial motility are required for pregnancy- and PP-associated adaptations of the maternal heart, iRhot1/2-KO mice were subjected to successive pregnancies and PP nursing periods. Immunoblotting confirmed the deletion of RHOT1 and RHOT2 in LV tissue from iRhot1/2-KO mice (Figure 1D). We previously reported that cardiomyocyte-selective *Rhot1/2* deletion during embryogenesis, driven by the αMHC *(Myh6)* promoter, results in perturbed sarcomere maturation and fatal cardiomyopathy [21]. The experimental design used in the present study allowed for physiological cardiac development prior to the induction of gene deletion in the adult heart. Motility of mitochondria isolated from iRhot1/2-KO hearts 8 weeks post-tamoxifen was impaired, as indicated by a reduced number of mobile mitochondria using an in vitro motility assay on surface-immobilized microtubules (Figure 1E,F), consistent with our previous findings [21]. To elucidate the contribution of mitochondrial motility to pregnancy- and PP-associated adaptations of the maternal heart, iRhot1/2-KO and control mice were analyzed after three to five successive pregnancies and PP nursing periods. Mice were sacrificed either post-delivery (time point delivery; D) or at the end of the PP nursing period, 18–21 days post-delivery (time point postpartum; PP). Age-matched nulliparous (NP) mice served as controls (Figure 1G).

**Figure 2:**
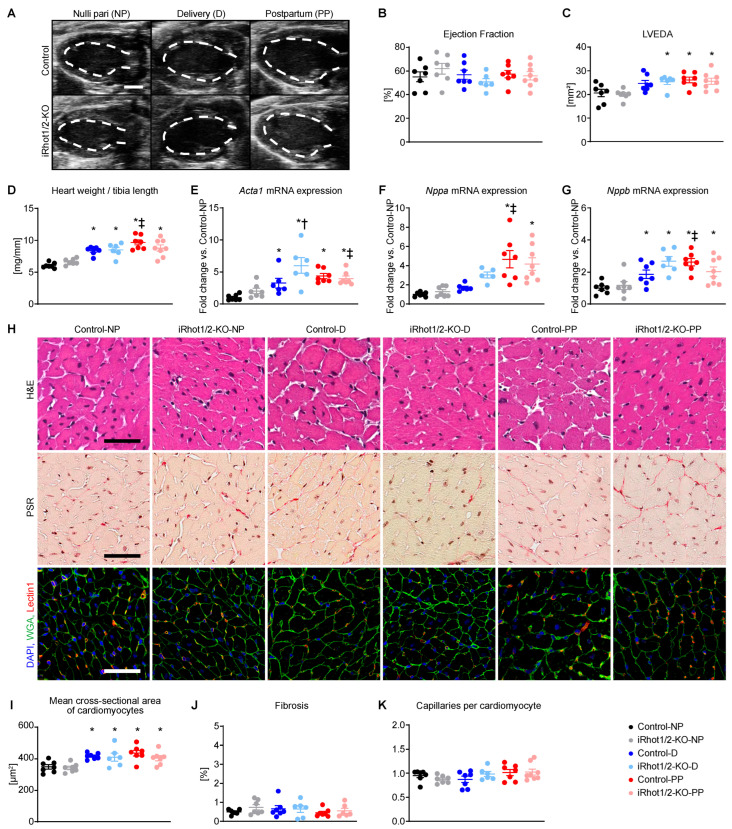
*Rhot1/2* expression is dispensable for cardiac structure and contractile function during pregnancy and postpartum. (**A**) Representative B-mode echocardiography images at end-diastole in long-axis projection from mice as indicated. The dashed line indicates left ventricular end-diastolic area (LVEDA), scale bars: 2 mm. (**B**) Ejection fraction (*n* = 6–8) and (**C**) LVEDA (*n* = 6–8; #) measured by transthoracic echocardiography. (**D**) Heart weights normalized to tibia length (*n* = 6–8; #). (**E**–**G**) mRNA expression of heart failure markers *Acta1* (#, $), *Nppa* (#), and *Nppb* (#, &) normalized to *Rps16* and presented as fold change vs. Control-NP (*n* = 5–8). (**H**) Representative Hematoxylin and Eosin (H&E), Picrosirius Red (PSR), and merged immunostainings showing DAPI (blue), wheat germ agglutinin (WGA, green), and Lectin 1 (red) to visualize capillaries (scale bars: 50 µm each). (**I**–**K**) Stereological quantification of (**I**) mean cross-sectional area of cardiomyocytes (#), (**J**) cardiac fibrosis, and (**K**) capillaries per cardiomyocyte (*n* = 6–8). Data are reported as mean values ± SEM. * *p* < 0.05 vs. nulliparous (NP) same genotype, † *p* < 0.05 vs. Control same time point, ‡ *p* < 0.05 vs. delivery (**D**) same genotype. Two-way ANOVA was performed to analyze differences by time point and genotype, followed by Holm–Šídák post hoc analysis (# *p* < 0.05 for time point, $ *p* < 0.05 for genotype, and & *p* < 0.05 for the interaction between time point and genotype). Control-D, Control/delivery; Control-NP, Control/nulliparous; Control-PP, Control/postpartum; iRhot1/2-KO-D, iRhot1/2-KO/delivery; iRhot1/2-KO-NP, iRhot1/2-KO/nulliparous; iRhot1/2-KO-PP, iRhot1/2-KO/postpartum.

Transthoracic echocardiography revealed no differences in contractile function between groups, as assessed by ejection fraction. LVEDA was increased in iRhot1/2-KO mice at D and PP relative to NP, whereas LVEDA was increased in controls only at PP relative to NP (Figure 2A–C, Supplementary Table S1). Heart weights normalized to tibia length were increased at D and PP relative to NP, independent of genotype (Figure 2D, Supplementary Table S2). mRNA expression of cardiac stress markers was induced relative to NP, independent of *Rhot1/2* expression. *Acta1* and *Nppb* were increased at D and PP, whereas *Nppa* expression was only increased at PP (Figure 2E–G). Stereological analysis of LV sections showed an increased mean cross-sectional area of cardiomyocytes at D and PP relative to NP, independent of genotype (Figure 2H,I). Interstitial fibrosis (Figure 2H,J) and capillary density (Figure 2H,K) did not differ between groups. Similarly, mRNA expression of *vascular endothelial growth factor A (Vegfa)*, a key mediator of angiogenesis [29], was relatively unchanged between groups (Supplementary Figure S1). Together, these data indicate that *Rhot1/2* expression is dispensable for cardiac structure and contractile function in the maternal heart during pregnancy and PP.

### 2.2. Rhot1/2-Mediated Mitochondrial Motility Is Dispensable for Mitochondrial Dynamics, Morphology, DNA Content, and Enzymatic Activity During Pregnancy and PP

**Figure 3:**
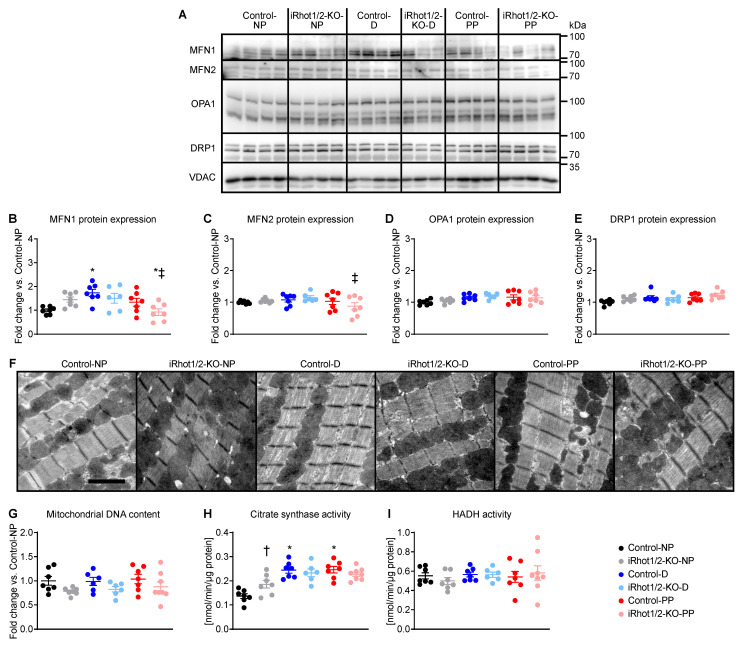
Deletion of *Rhot1* and *Rhot2* does not affect mitochondrial dynamics, morphology, DNA content, and enzymatic activity during pregnancy and postpartum. (**A**) Representative immunoblots of left ventricular homogenates and densitometric quantification of (**B**) MFN1 (#, &), (**C**) MFN2, (**D**) OPA1 (#), and (**E**) DRP1 (#) normalized to VDAC and presented as fold change vs. Control-NP (*n* = 6–7). (**F**) Representative electron micrographs of left ventricular tissue (scale bars: 2 µm). (**G**) Mitochondrial DNA content in left ventricular tissue ($; *n* = 6–8). (**H**,**I**) Citrate synthase (#, &) and hydroxyacyl-coenzyme A dehydrogenase (HADH) enzymatic activity in left ventricular tissue (*n* = 6–8). Data are reported as mean values ± SEM. * *p* < 0.05 vs. nulliparous (NP) same genotype, † *p* < 0.05 vs. Control same time point, ‡ *p* < 0.05 vs. delivery (**D**) same genotype. Two-way ANOVA was performed to analyze differences by time point and genotype, followed by Holm–Šídák post hoc analysis (# *p* < 0.05 for time point, $ *p* < 0.05 for genotype, and & *p* < 0.05 for the interaction between time point and genotype). Control-D, Control/delivery; Control-NP, Control/nulliparous; Control-PP, Control/postpartum; iRhot1/2-KO-D, iRhot1/2-KO/delivery; iRhot1/2-KO-NP, iRhot1/2-KO/nulliparous; iRhot1/2-KO-PP, iRhot1/2-KO/postpartum.

Mitochondria are dynamic organelles that constantly undergo fusion and fission processes, i.e., mitochondrial dynamics, which are mediated by mitofusin (MFN) 1, MFN2, optic atrophy 1 (OPA1), and dynamin-related protein 1 (DRP1). MFN1, MFN2, OPA1, and DRP1 are highly expressed in the heart, and their genetic deletion in murine models results in mitochondrial dysfunction and heart failure [30,31]. Moreover, RHOT proteins interact with MFN1/2, as reported for neurons [32,33]. We hypothesized that RHOT proteins preserve mitochondrial dynamics and structure in the maternal heart in the context of increased workload during pregnancy and PP. Expression of proteins involved in mitochondrial fusion/fission (Figure 3A–E), mitochondrial morphology as assessed by electron micrographs (Figure 3F), and mitochondrial DNA content (Figure 3G) were relatively unchanged between groups. Interestingly, *Rhot1/2* deletion increased citrate synthase enzymatic activity under basal conditions, as indicated by the comparison iRhot1/2-KO-NP vs. Control-NP. In controls, citrate synthase activity increased at D and PP relative to NP, while no difference was detected between controls and iRhot1/2-KO at D and PP (Figure 3H). Hydroxyacyl-coenzyme A dehydrogenase (HADH) enzymatic activity did not differ between groups (Figure 3I). Oxidative stress was assessed by 4-hydroxynonenal (4-HNE) and mitochondrial superoxide dismutase (MnSOD) protein expression. 4-HNE protein expression was increased at D and PP independent of *Rhot1* and *Rhot2* expression. Interestingly, MnSOD expression did not differ between groups (Supplementary Figure S2). Together, these data demonstrate that RHOT protein expression in cardiomyocytes does not affect mitochondrial dynamics, morphology, DNA content, oxidative stress, and citrate synthase and HADH enzymatic activity during pregnancy and PP.

### 2.3. Mitochondrial Motility Preserves Cardiac Energetic and Contraction Gene Expression in Maternal Hearts During Pregnancy and PP

During pregnancy, metabolism of the maternal heart switches from primarily glucose oxidation toward fatty acid oxidation [34]. Similarly, fatty acid oxidation increases in the postnatal heart in the context of increased workload, accompanied by decreased glucose oxidation, mitophagic replacement of fetal mitochondria, and transcriptional reprogramming of metabolic genes [35]. Damaged mitochondria are engulfed by autophagosomes, which subsequently fuse with lysosomes for degradation, a process that depends on local mitochondrial distribution. We therefore sought to test the hypothesis that mitochondrial motility, mediated by RHOT proteins, is required for mitophagic replacement of mitochondria, metabolic adaptations, and transcriptional reprogramming of cardiac substrate oxidation genes in the maternal heart during pregnancy and PP. Gene expression profiling was performed by RNA sequencing using LV tissue. A total of 19,360 detected transcripts were considered for analysis (Supplementary Table S3).

**Figure 4:**
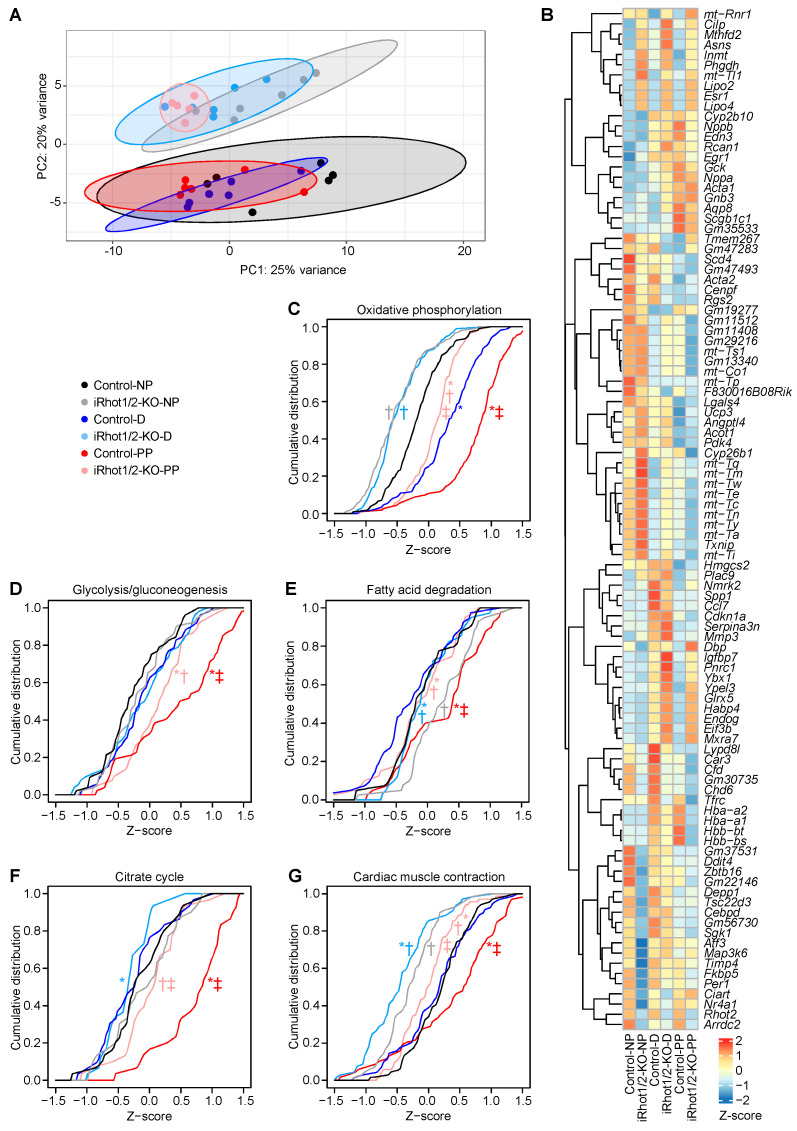
Gene expression during pregnancy and postpartum as determined by RNA sequencing. (**A**) Principal component analysis to visualize global gene expression clusters by time point and genotype (*n* = 6). (**B**) Heatmap of RNA sequencing count data corresponding to the 100 genes with the greatest variance across groups. Data are clustered by row after applying the regularized log transformation in DESeq2. Color intensity indicates row-scaled Z-scores. (**C**–**G**) Cumulative distribution plots for Z-scores of (**C**) oxidative phosphorylation, (**D**) glycolysis/gluconeogenesis, (**E**) fatty acid degradation, (**F**) citrate cycle, and (**G**) cardiac muscle contraction genes based on KEGG pathways. A rightward shift indicates an increase in the abundance of pathway genes. * *p* < 0.05 vs. nulliparous (NP) same genotype, † *p* < 0.05 vs. Control same time point, ‡ *p* < 0.05 vs. delivery (**D**) same genotype (two-sided Kolmogorov–Smirnov test). Control-D, Control/delivery; Control-NP, Control/nulliparous; Control-PP, Control/postpartum; iRhot1/2-KO-D, iRhot1/2-KO/delivery; iRhot1/2-KO-NP, iRhot1/2-KO/nulliparous; iRhot1/2-KO-PP, iRhot1/2-KO/postpartum.

Principal component analysis (PCA) revealed that global gene expression was relatively similar between time points in mice of the same genotype, with a predominant effect of *Rhot1/2* deletion on gene expression (Figure 4A). The heatmap (Figure 4B) displays the top 100 genes with the greatest variance across groups and shows differentially regulated transcripts associated with mitochondria, metabolism, and cardiac stress. We next investigated differences in gene expression using pairwise comparisons between groups. We explored nine contrasts representing differences in time points for controls (contrasts I-III), differences in time points for iRhot1/2-KO (contrasts IV-VI), and differences between iRhot1/2-KO and control mice at the same time point (contrasts VII-IX; Supplementary Figure S3 and Supplementary Table S3). We identified a total of 125 transcripts that were upregulated and 123 that were downregulated in iRhot1/2-KO-NP vs. Control-NP (contrast VII), 690 upregulated and 635 downregulated in iRhot1/2-KO-D vs. Control-D (contrast VIII), and 705 upregulated and 587 downregulated in iRhot1/2-KO-PP vs. Control-PP (contrast IX; cutoff: FDR < 0.1 each; Supplementary Figure S3G–I and Supplementary Table S3). These data reveal that *Rhot1/2* expression has a greater impact on gene expression at D and PP relative to NP. Ingenuity Pathway Analysis (IPA) identified integrin-linked kinase (ILK) signaling as the top canonical pathway in contrast VII (iRhot1/2-KO-NP vs. Control-NP) and mitochondrial dysfunction as the top canonical pathway in contrast VIII (iRhot1/2-KO-D vs. Control-D; cutoff: FDR < 0.1; Supplementary Table S4). Expression of *Stat3* and *Erbb4*, which are critical for peripartum-associated heart failure [13,14], was not different in iRhot1/2-KO relative to controls at the time points investigated (cutoff: FDR < 0.1; Supplementary Table S3).

We next focused our analysis on transcripts associated with cardiac energetics and contraction. Cumulative distribution analysis showed an increase in oxidative phosphorylation gene expression in controls at D relative to NP with a further induction at PP. iRhot1/2-KO exhibited decreased abundance of oxidative phosphorylation genes relative to controls at the time points investigated (Figure 4C and Supplementary Figure S4A). Expression of genes associated with glycolysis/gluconeogenesis (Figure 4D and Supplementary Figure S4B), fatty acid degradation (Figure 4E and Supplementary Figure S4C), and citrate cycle (Figure 4F and Supplementary Figure S4D) increased in Control-PP relative to Control-NP, while no difference was detected for Control-D relative to Control-NP. Interestingly, expression of genes related to fatty acid degradation was increased in iRhot1/2-KO-NP and iRhot1/2-KO-D relative to controls at the same time point, but decreased in iRhot1/2-KO-D and iRhot1/2-KO-PP relative to iRhot1/2-KO-NP (Figure 4E and Supplementary Figure S4C). Importantly, expression of glycolysis/gluconeogenesis, fatty acid degradation, and citrate cycle genes was decreased in iRhot1/2-KO-PP relative to Control-PP (Figure 4D–F and Supplementary Figure S4B–D). The switch in cardiac substrate oxidation during pregnancy toward fatty acid oxidation results from induction of pyruvate dehydrogenase kinase 4 (PDK4) [34]. PDK4 phosphorylates and inhibits pyruvate dehydrogenase (PDH) activity, which decreases the conversion of pyruvate, the end product of glycolysis, into Acetyl-CoA [36]. Interestingly, PDK4 protein expression increased in both iRhot1/2-KO and control mice at D, but not at PP (Supplementary Figure S5A,B). *Ppargc1a* transcript levels were similar between groups (Supplementary Figure S5C). Protein expression of the mitophagy marker BNIP3 increased independent of *Rhot1/2* expression at D and PP (Supplementary Figure S6A,B). LC3-II expression, normalized to both vinculin and LC3-I, was not different between groups (Supplementary Figure S6A,C,D). PTEN-induced putative kinase 1 (PINK1) is a mitochondrial serine/threonine kinase involved in the regulation of mitochondrial quality control. Upon mitochondrial damage, PINK1 accumulates on the outer mitochondrial membrane and phosphorylates the E3 ubiquitin ligase Parkin, promoting Parkin recruitment to mitochondria and initiation of mitophagy [37,38]. In addition, autophosphorylation of PINK1 at Ser228 contributes to Parkin recruitment and mitophagy activation [38,39]. Interestingly, PINK1 phosphorylation at Ser228 was not different between groups, suggesting that deletion of *Rhot1/2* does not affect this specific regulatory modification of PINK1 at the time points investigated (Supplementary Figure S6A,E). Thus, RHOT proteins mediate a transcriptional program that maintains cardiac energetic gene expression during pregnancy and PP, independent of mitophagy and *Ppargc1a* expression.

Our previous studies identified a critical role for RHOT proteins in cardiomyocyte sarcomere maturation in the context of the postnatal increase in cardiac workload [21]. Similar to postnatal adaptations, the cardiac workload of the maternal heart increases during pregnancy [4,10]. We therefore hypothesized that RHOT proteins maintain sarcomere structure and contraction gene expression in the maternal heart during pregnancy and PP. The expression of genes associated with cardiac muscle contraction increased in controls at PP compared with NP (Figure 4G and Supplementary Figure S4E). Similar to oxidative phosphorylation genes, the expression of cardiac muscle contraction genes was reduced in iRhot1/2-KO relative to controls at the time points investigated. In summary, these data identify that RHOT proteins preserve cardiac energetic and contraction gene expression in maternal hearts during pregnancy and PP.

## 3. Discussion

The maternal heart enlarges to maintain circulatory flow during pregnancy, compensating for the increased workload and supporting fetal growth [1]. Previous studies showed a critical role for motor protein-driven mitochondrial motility and RHOT proteins in the context of increased workload, cardiomyocyte hypertrophy, and sarcomere maturation [21,25,26,27]. The present study demonstrates that RHOT proteins are not required for cardiac structure and hypertrophy, mitochondrial dynamics, mitochondrial enzymatic activity, and mitochondrial DNA content in the murine maternal heart during pregnancy and PP. Despite repression of a transcriptional program by which RHOT proteins preserve cardiac energetic and contraction gene expression, loss of *Rhot1/2* expression in the adult heart does not lead to peripartum-associated heart failure.

Our previous studies revealed a critical role for RHOT proteins in sarcomere maturation, as evidenced by fatal cardiomyopathy in mice with cardiomyocyte-selective *Rhot1/2* deletion during embryogenesis [21]. In this model, Cre/loxP-mediated gene deletion was driven by the αMHC promoter, which is expressed as early as embryonic day 9.5 [40]. The underlying mechanisms resulting in fatal cardiomyopathy comprise decreased binding of RHOT1/2-deficient mitochondria to contractile muscle fiber proteins and disrupted mitochondrial positioning, which impairs local ATP production and sarcomere maturation [21]. The mammalian heart is characterized by dramatic postnatal hypertrophy. For example, the human heart increases more than 10-fold in weight during postnatal maturation, from an average of 20–25 g in newborns to ~300 g in adults. In contrast, LV mass increases during pregnancy by approximately 30% [2,3]. Even though our study reveals that *Rhot1* and *Rhot2* mRNA expression is increased in murine WT hearts during pregnancy and PP, loss of RHOT proteins does not lead to peripartum-associated heart failure. Thus, RHOT proteins are not required for cardiac structure and contractile function in the adult heart in the absence of a strong hypertrophic stimulus, as supported by our present and previous studies reporting no differences in iRhot1/2-KO hearts relative to controls [21]. Moreover, mice overexpressing *Rhot2* exhibit attenuated pressure overload-induced heart failure following transverse aortic constriction, using a protocol that results in approximately a 2-fold increase in heart weight in controls [27]. Together, these studies indicate that RHOT protein expression is required for cardiac structure and contractile function under conditions of pronounced cardiomyocyte hypertrophy; however, it is dispensable for cardiomyocyte ultrastructure and contractility during pregnancy- and PP-associated adaptations of the maternal heart, which are characterized by relatively mild hypertrophy.

*Ppargc1a* regulates angiogenesis in the heart and transgenic mice with cardiomyocyte-selective deletion of *Ppargc1a* develop PPCM that can be rescued by pro-angiogenic therapy [12]. Interestingly, the present study showed no differences in *Ppargc1a* mRNA expression and capillary density between groups. Similarly, *Vegfa* mRNA expression, which is regulated by *Ppargc1a* [12], was not altered at the time points investigated. Cardiomyocyte-selective *Stat3* deletion results in PPCM, which is associated with increased hypertrophy, fibrosis, and decreased capillary density [13]. STAT3 is also expressed in mitochondria [41] and was not different in iRhot1/2-KO hearts relative to controls at the time points investigated. Together, these studies demonstrate that *Ppargc1a* and *Stat3* expression is required for peripartum-associated adaptations of the maternal heart [12,13]. However, their expression is not upregulated to compensate for the increased cardiac workload in the experimental setting involving successive pregnancies and PP nursing periods used in the present study. Our RNA sequencing experiment identified a transcriptional program mediated by RHOT proteins that preserves cardiac energetic and metabolic gene expression during pregnancy and PP. Metabolic transcripts are increased in cardiac tissue from pregnant mice [28]. Furthermore, substrate oxidation in the maternal heart switches from primarily glucose oxidation to fatty acid oxidation driven by increased PDK4 expression [34]. Similarly, we observed increased PDK4 expression at delivery, but not at PP, which was independent of *Rhot1/2* expression. We analyzed cardiac tissue after three to five pregnancies, whereas previous murine studies have examined maternal hearts during or after the first pregnancy [28,34]. The experimental design of the present study better reflects the increased risk for PPCM in subsequent pregnancies and allows investigation of the impact of RHOT proteins on mitophagy during successive pregnancies and PP nursing periods. The increase in BNIP3 protein expression at D and PP, which is independent of *Rhot1/2* expression, suggests that RHOT proteins and mitochondrial motility are dispensable for mitophagy during pregnancy- and PP-associated adaptations. This is supported by no difference in PINK1 phosphorylation at Ser228, which regulates mitophagy activation [38,39].

The contribution of perturbed substrate oxidation to the development of PPCM is highlighted by the impaired metabolic flexibility of cardiomyocytes from PPCM patients [16]. Our gene profiling experiment showed that RHOT proteins preserve cardiac energetic and contraction gene expression in maternal hearts independent of *Ppargc1a*. These data are consistent with our previous studies in mice with embryonic cardiomyocyte-selective deletion of *Rhot1/2* that exhibit a loss of sarcomere structure [21]. The underlying mechanisms by which RHOT proteins regulate cardiac energetic and structure gene expression remain to be determined and will be the subject of future studies.

Limitations of the present study include that contractile function was assessed using two-dimensional transthoracic echocardiography, which might not detect subtle differences in contractile function between groups that may exist and could only be detected by invasive hemodynamic measurements. Cardiomyocyte-level functional analyses, such as calcium handling and single-cell contractility, were not performed and may provide further mechanistic insight in future studies. Importantly, *Rhot1/2* deletion had no impact on the mRNA expression of heart failure markers. These data suggest no difference in hemodynamic stress in iRhot1/2-KO hearts relative to controls at the time points investigated. Our RNA sequencing experiment was performed in LV tissue, which contains different cell types, including cardiomyocytes, endothelial cells, and fibroblasts. Therefore, we cannot determine the impact of inducible cardiomyocyte-selective *Rhot1/2* deletion on the various cellular populations within the heart. Another limitation of this study is that a comprehensive assessment of mitochondrial function, including respiratory chain complex activity, membrane potential, and ROS production, was not performed. However, the combination of biochemical, molecular, and ultrastructural analyses provides a robust overview of mitochondrial integrity under the experimental conditions, suggesting that *Rhot1/2* deletion does not affect mitochondrial integrity or function during pregnancy and postpartum. These findings are further supported by previous studies demonstrating preserved mitochondrial respiratory capacity in isolated mitochondria from iRhot1/2-KO hearts under non-stressed conditions [21]. The present study was conducted in mice with inducible cardiomyocyte-selective deletion of both *Rhot1* and *Rhot2* (iRhot1/2-KO). *Rhot1* and *Rhot2* share a sequence homology of 60% in mammals [20]. Based on our results from iRhot1/2-KO mice, it is unlikely that transgenic mice with inducible cardiomyocyte-selective deletion of *Rhot1* or *Rhot2* in the adult heart would show a different phenotype compared to iRhot1/2-KO. This is supported by our previous studies, which demonstrated similar phenotypes in transgenic mice with embryonic cardiomyocyte-selective deletion of *Rhot1*, *Rhot2*, or both [21].

**Figure 5:**
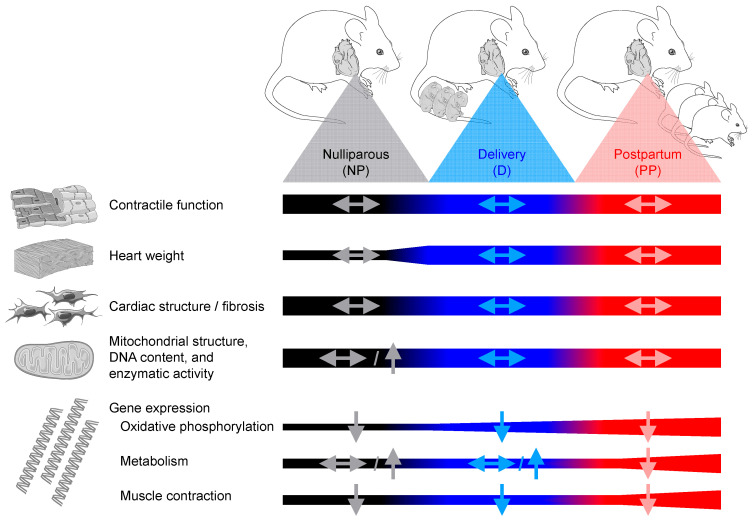
Summary of RHOT protein-mediated adaptations of maternal hearts during pregnancy and postpartum. Data obtained after three to five deliveries are summarized relative to nulliparous controls. Bars indicate changes in control hearts over time, and arrows indicate changes in iRhot1/2-KO hearts relative to controls expressing *Rhot1* and *Rhot2* at the same time point.

Together, the present study reveals that RHOT proteins are dispensable for contractile function, cardiomyocyte ultrastructure, angiogenesis, and mitochondrial adaptations in the maternal heart during pregnancy- and PP-associated adaptations. Furthermore, we identified a transcriptional program by which RHOT proteins preserve cardiac energetic and contraction gene expression during pregnancy and PP, independent of mitophagy. Thus, loss of *Rhot1/2* expression in the adult heart does not lead to peripartum-associated heart failure, despite reduced expression of cardiac energetic and contraction genes (Figure 5).

## 4. Materials and Methods

### 4.1. Animals

Experiments were performed in accordance with protocols approved by local state authorities (Niedersächsisches Landesamt für Verbraucherschutz und Lebensmittelsicherheit; protocol numbers: 18/3058 and 22/00043). As the present study investigated pregnancy- and PP-associated adaptations of the maternal heart, only female mice were used. Animals were housed in cages supplemented with nesting material with a 12-h light/12-h dark cycle with ad libitum access to food and water. Animal well-being was assessed using score sheets with predefined criteria, including breathing, coat condition, mobility, and response to stimuli. Mice were randomly assigned to the experimental groups after weaning based on cage allocation by the animal care staff. For tissue harvest, mice were euthanized by cervical dislocation under isoflurane anesthesia.

#### 4.1.1. Wildtype Mice

Female WT C57/BL6J mice were mated at 8 weeks of age and separated from males after pregnancy was confirmed by visual inspection. WT mice were sacrificed 1 day post-delivery (time point delivery; WT-D) or at the end of the PP nursing period, 18–21 days post-delivery (time point postpartum; WT-PP), following the first pregnancy. Age-matched nulliparous WT (WT-NP) mice served as controls.

#### 4.1.2. Transgenic Mice

Mice with inducible cardiomyocyte-selective deletion of *Rhot1* and *Rhot2* (iRhot1/2-KO; *Rhot1*^lox/lox^ *Rhot2*^lox/lox^ αMHC-MCM^+/-^) were generated and genotyped as previously described [21]. Cre-loxP recombination in iRhot1/2-KO hearts was mediated by the tamoxifen-inducible αMHC-MerCreMer (MCM) transgene [42]. Control mice had the genotype *Rhot1*^lox/lox^ *Rhot2*^lox/lox^. Both iRhot1/2-KO and control mice were intraperitoneally injected with tamoxifen (0.5 mg/day) starting at 7–9 weeks of age for five consecutive days (Sigma Aldrich, St. Louis, MO, USA). All time points reported refer to the first tamoxifen injection. iRhot1/2-KO and control mice were mated 2–3 weeks after the final tamoxifen injection. iRhot1/2-KO and control mice were sacrificed one or two days post-delivery (time point delivery; D) or at the end of the nursing period, 18–21 days post-delivery (time point postpartum; PP). iRhot1/2-KO and control mice were separated from male mice after pregnancy was confirmed to prevent overlapping pregnancy and nursing. Data for the D and PP groups were collected after three to five pregnancies. Age-matched NP mice were investigated at a mean age of 43 weeks.

### 4.2. Immunoblotting Analysis

Protein extraction, immunoblotting, membrane stripping, and densitometric analysis were performed as previously described [21,43]. Primary and secondary antibodies are listed in Supplementary Table S5.

### 4.3. Measurement of Mitochondrial Motility

Mitochondria were isolated and in vitro mitochondrial motility was assessed on surface-immobilized Cy5-labeled microtubules as previously described [21].

### 4.4. Transthoracic Echocardiography

Transthoracic echocardiography was performed under isoflurane anesthesia (induction at 5% and maintenance at 2% isoflurane) using an MX400 transducer coupled to a Vevo 3100 echocardiograph (VisualSonics Inc., Toronto, ON, Canada). Mice assigned to the time point D were examined two days prior to the estimated delivery, whereas mice assigned to the time point PP were examined at the end of the nursing period. Mice were placed in the supine position on a heating pad (37 °C), and eye care solution was applied to prevent corneal injury. Two-dimensional B-mode images were acquired, and endocardial silhouettes were traced manually. Ejection fraction was determined in the long-axis projection using the VevoStrain 2.0 software (VisualSonics Inc., Toronto, ON, Canada). LV end-diastolic area (LVEDA) and LV end-systolic area (LVESA) were recorded from the long-axis parasternal view. Fractional area change (%) was calculated as [(LVEDA − LVESA)/LVEDA] * 100.

### 4.5. Histological and Immunohistochemical Analysis

Cardiac tissue was fixed in 4% buffered formaldehyde solution, embedded in paraffin, cut into 2-µm thick sections, and stained with Hematoxylin and Eosin (H&E) or Picrosirius Red (PSR) solution. Slides were scanned using a slide scanner (Aperio CS2, Leica Biosystems, Wetzlar, Germany) [43]. The fraction of fibrotic tissue was quantified in PSR-stained sections using Adobe Photoshop CS5 software (Adobe, San Jose, CA, USA) [44]. For immunohistochemical analyses, sections were prepared and stained with wheat germ agglutinin (WGA; dilution 1:500; Thermo Fisher Scientific, Waltham, MA, USA), Lectin 1 (dilution 1:50; Biozol, Eching, Germany), and DAPI (Zytomed Systems GmbH, Berlin, Germany) as previously described [43]. Images were scanned and analyzed as previously described [43].

### 4.6. Electron Microscopy

Preparation and imaging of transmission electron microscopy samples from LV tissue were performed as previously reported [21].

### 4.7. Measurement of Mitochondrial DNA Content

Mitochondrial DNA (mtDNA) content in LV tissue was determined as previously described [43]. The mtDNA-to-nuclear DNA ratio was measured using the following primers: mtDNA-encoded *cytochrome c oxidase 1* (forward: 5′-ACTATACTACTAACAGACCG-3′; reverse: 5′-GGTTCTTTTTTTCCGGAGTA-3′) and nuclear DNA-encoded *cyclophilin A* (forward: 5′-ACACGCCATAATGGCACTGG-3′; reverse: 5′-CAGTCTTGGCAGTGCAGAT-3′).

### 4.8. Mitochondrial Enzymatic Activity Assays

Citrate synthase and HADH activities in LV tissue were measured using a Synergy™ HT multi-detection microplate reader (BioTek Instruments, Winooski, VT, USA) in a total reaction volume of 200 μL, as previously described [45].

### 4.9. RNA Sequencing

Total RNA from LV tissue was isolated using the NucleoSpin RNA kit (Macherey-Nagel, Düren, Germany). RNA quality and integrity were assessed using a 2100 Bioanalyzer (Agilent Technologies, Waldbronn, Germany). The RNA sequencing library was generated from 20 ng of total RNA using the NEBNext^®^ Single Cell/Low Input Library Prep Kit (New England BioLabs, Ipswich, MA, USA) according to the manufacturer’s instructions. Libraries were sequenced on a NovaSeq 6000 system using the NovaSeq 6000 S1 Reagent Kit (Illumina, San Diego, CA, USA; 100 cycles, paired end run) with an average of 5 * 10^7^ reads per RNA sample (Illumina, San Diego, CA, USA). Quality reports were generated for each FASTQ file using FastQC (http://www.bioinformatics.babraham.ac.uk/projects/fastqc; accessed on 2 April 2026). Raw FASTQ sequences were trimmed for base-call quality and adapter contamination using the ea-utils fastq-mcf tool, and reads shorter than 15 bp were removed. Trimmed reads were aligned to the murine reference genome using the open-source short-read aligner STAR, with parameters set according to the log file [46]. RNA sequencing data analysis was performed using the statistical programming language R (v4.1.1) [47,48]. Feature counts were determined using the R package Rsubread (v2.2.6) [49]. Transcript annotation was performed using the R package biomaRt (v2.44.4) [50], and differential gene expression was evaluated using the R package DESeq2 (v1.32.0) [51]. Only transcripts with a total read count ≥10 in six samples (corresponding to the group size) were considered for further analysis. For comparison of transcript expression profiles, mRNA transcripts were classified according to KEGG pathways: cardiac muscle contraction (mmu04260), citrate cycle (mmu00020), fatty acid degradation (mmu00071), glycolysis/gluconeogenesis (mmu00010), and oxidative phosphorylation (mmu00190; https://www.genome.jp/kegg/; accessed on 2 April 2026). Cumulative distribution analysis was performed using R, and *p*-values are reported for two-sided Kolmogorov–Smirnov tests (significance threshold: *p* < 0.05). Functional analysis was performed using the Ingenuity Pathway Analysis (IPA) tool (QIAGEN Inc., Germantown, MD, USA; https://www.qiagen.com/us/products/discovery-and-translational-research/next-generation-sequencing/informatics-and-data/interpretation-content-databases/ingenuity-pathway-analysis/; accessed on 2 April 2026). RNA sequencing data were deposited into the Gene Expression Omnibus database (https://www.ncbi.nlm.nih.gov/geo/; accessed on 2 April 2026), accession number: GSE328619.

### 4.10. Quantitative RT-PCR Analysis

RNA extraction, cDNA synthesis, and quantitative real-time PCR analysis were performed as previously reported [44]. Primer sequences are listed in Supplementary Table S6.

### 4.11. Statistics

Data are expressed as mean ± SEM. Whenever possible, investigators were blinded to group allocation throughout the experiments and subsequent analyses. Only mice surviving to the predefined tissue harvest time point were included in further analyses. Sample size for murine studies was determined based on our previous experience with murine heart failure models and RNA sequencing-based gene profiling [21,43,44,48]. GraphPad Prism 8 software (GraphPad Software, San Diego, CA, USA) was used for statistical analysis. Unpaired two-tailed Student’s *t*-tests were used for comparisons between two groups. Multi-group comparisons were performed using ANOVA followed by Holm–Šídák post hoc tests. A *p*-value of <0.05 was considered significantly different.

## Data Availability

RNA sequencing data were deposited into the Gene Expression Omnibus database (https://www.ncbi.nlm.nih.gov/geo/; accessed on 21 April 2026), accession number: GSE328619.

## Supplementary Materials

The following supporting information can be downloaded at: https://www.mdpi.com/article/10.3390/ijms27114991/s1.
