# Supplementary material for: Peripartum-Associated Heart Failure Develops Independently of RHOT Proteins
Source: Int J Mol Sci. 2026 May 30;27(11):4991. doi: 10.3390/ijms27114991 (PMC13256580; doi:10.3390/ijms27114991)
Supplement: Supplementary file 1 [file ijms-27-04991-s001.zip › Supplementental Material.pdf]

SUPPLEMENTAL MATERIAL

FOR

Peripartum-Associated Heart Failure Develops Independently of RHOT Proteins

Natali Froese[1], Eluiesa Sina[1], Paolo Galuppo[1], Christopher Werlein[2], Anna Gigina[1], Jan Hegermann[3], Robert Geffers[4], Tim Scholz[5], Jan C. Kamp[6,7], Lavinia Neubert[2,7], Johanna Schneider[8], Melanie Ricke-Hoch[1], Alexander Dietl[9], Johann Bauersachs[1], Christian Riehle[1]

[1] Department of Cardiology and Angiology, Hannover Medical School, 30625 Hannover, Germany
[2] Institute of Pathology, Hannover Medical School, 30625 Hannover, Germany
[3] Research Core Unit Electron Microscopy, Institute of Functional and Applied Anatomy, Hannover Medical School, 30625 Hannover, Germany
[4] Research Group Genome Analytics, Helmholtz Center for Infection Research, 38124 Braunschweig, Germany
[5] Institute of Molecular and Cell Physiology, Hannover Medical School, 30625 Hannover, Germany
[6] Department of Respiratory Medicine and Infectious Diseases, Hannover Medical School, 30625 Hannover, Germany
[7] Biomedical Research in Endstage and Obstructive Lung Disease Hannover (BREATH), German Center for Lung Research (DZL), 30625 Hannover, Germany
[8] Department of Medicine IV, Medical Center – University of Freiburg, 79106 Freiburg, Germany
[9] Department of Internal Medicine II, University Hospital Regensburg, 93053 Regensburg, Germany

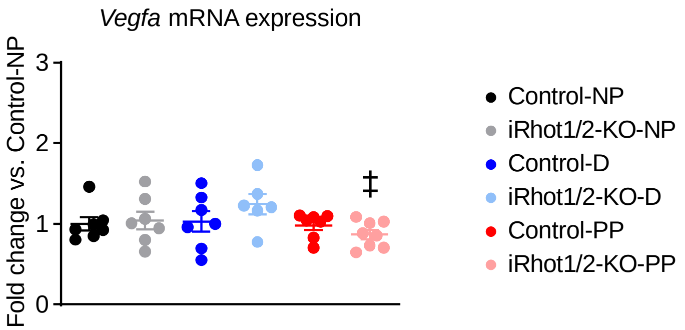

Figure S1. *Vegfa mRNA expression.*
Vascular endothelial growth factor A (Vegfa) mRNA expression in left ventricular tissue normalized to *Rps16* and presented as fold change vs. Control-NP (n=6-8). Data are reported as mean values ± SEM. ‡ $p<0.05$ vs. delivery (D) same genotype. Control-D, Control / delivery; Control-NP, Control / nulliparous; Control-PP, Control / postpartum; iRhot1/2-KO-D, iRhot1/2-KO / delivery; iRhot1/2-KO-NP, iRhot1/2-KO / nulliparous; iRhot1/2-KO-PP, iRhot1/2-KO / postpartum.

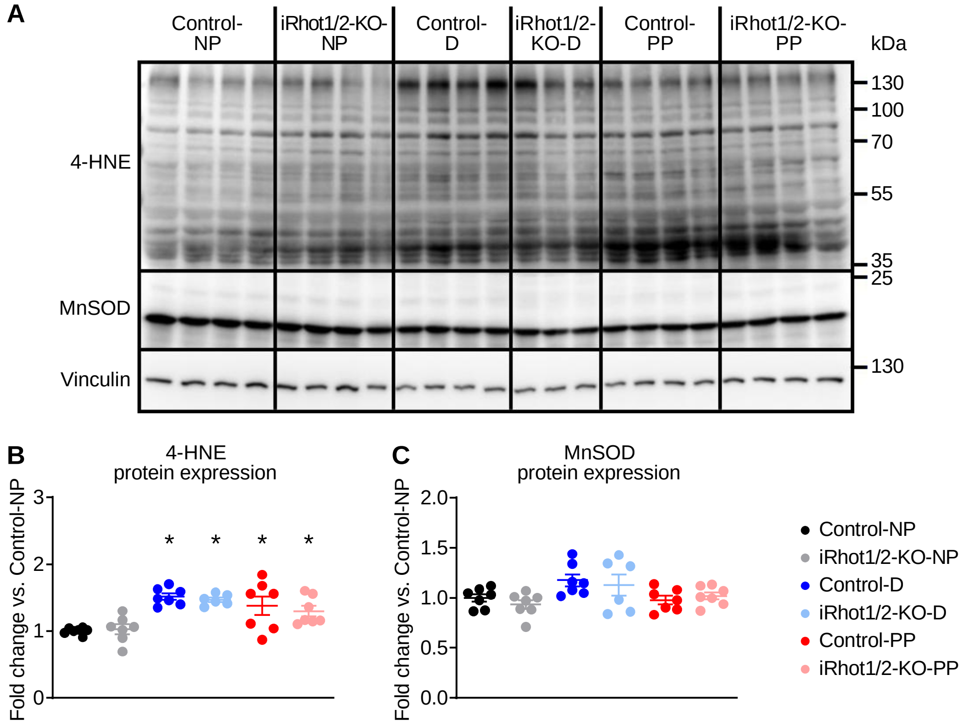

Figure S2. *Deletion of Rhot1 and Rhot2 does not affect markers of oxidative stress during pregnancy and postpartum.*

(A) Representative immunoblots of left ventricular homogenates and densitometric quantification of (B) 4-HNE (#) and (C) MnSOD (#) normalized to vinculin and presented as fold change vs. Control-NP (n=6-7). Data are reported as mean values ± SEM. * $p<0.05$ vs. nulliparous (NP) same genotype. Two-way ANOVA was performed to analyze differences by time point and genotype, followed by Holm-Šídák post hoc analysis (# $p<0.05$ for time point). Control-D, Control / delivery; Control-NP, Control / nulliparous; Control-PP, Control / postpartum; iRhot1/2-KO-D, iRhot1/2-KO / delivery; iRhot1/2-KO-NP, iRhot1/2-KO / nulliparous; iRhot1/2-KO-PP, iRhot1/2-KO / postpartum.

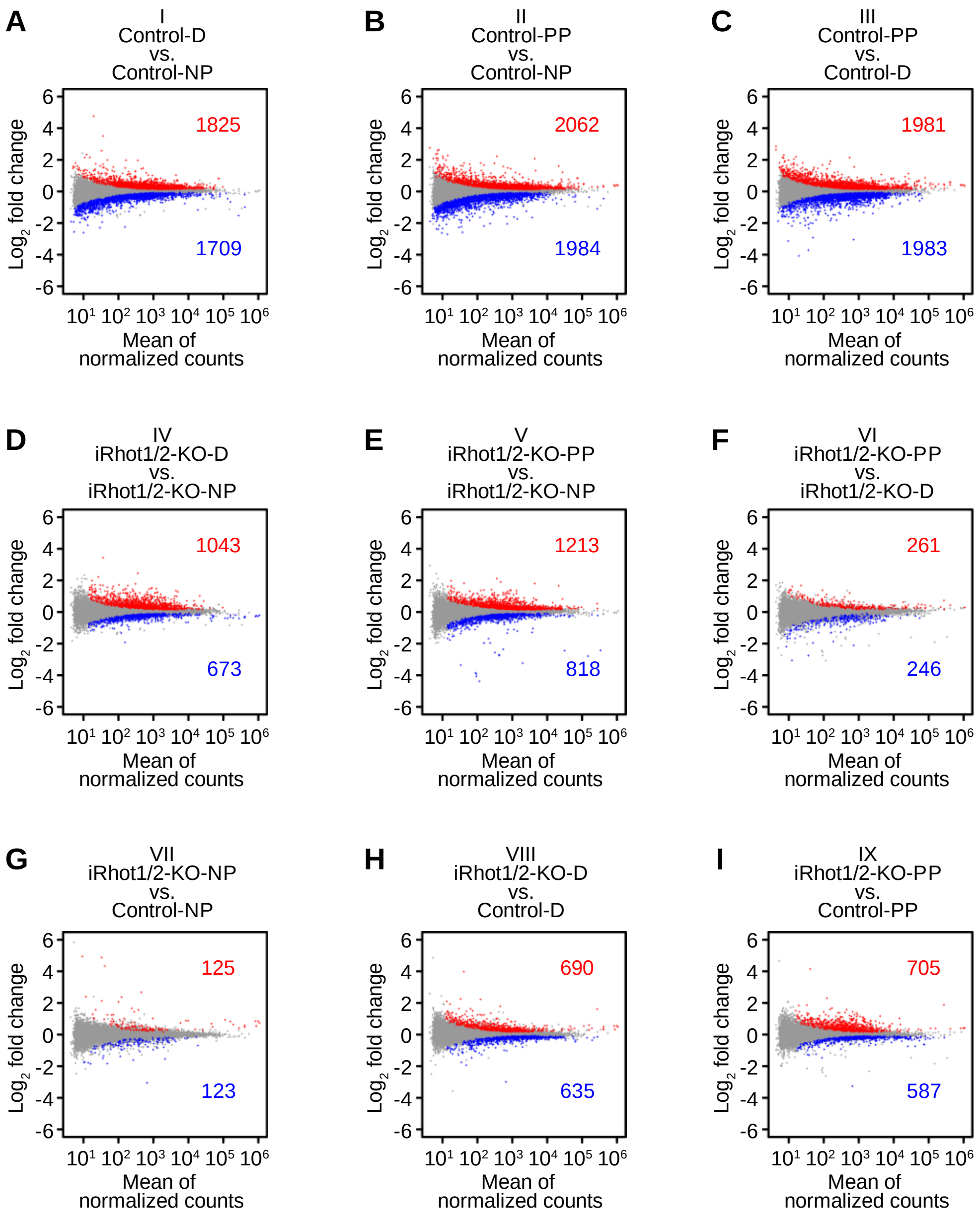

Figure S3. *MA plots for pairwise comparisons of gene expression analysis by RNA sequencing.* (A-I) Gene expression is presented as MA plots showing $\log_2$ fold change vs. mean of normalized counts for comparisons as indicated. Each cross represents one gene. Red and blue crosses indicate differentially up- or downregulated genes, and grey crosses indicate genes that are not differentially regulated genes for comparisons as indicated (cutoff: FDR<0.1; n=6). Control-D, Control / delivery; Control-NP, Control / nulliparous; Control-PP, Control / postpartum; iRhot1/2-KO-D, iRhot1/2-KO / delivery; iRhot1/2-KO-NP, iRhot1/2-KO / nulliparous; iRhot1/2-KO-PP, iRhot1/2-KO / postpartum.

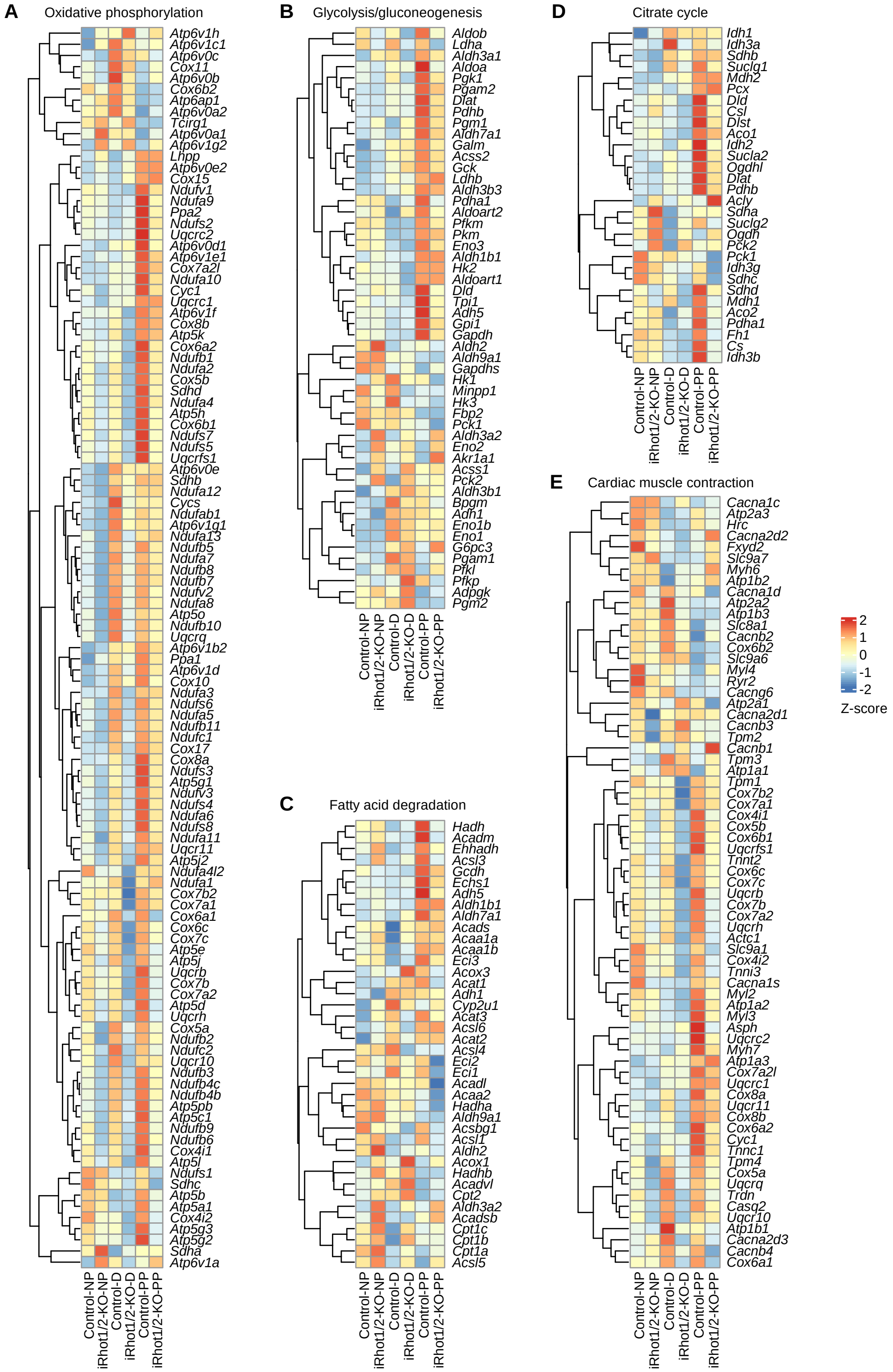

Figure S4. *Heatmap of Z-scores of KEGG pathways.*

(A-E) Heatmap of Z-scores of (A) oxidative phosphorylation, (B) glycolysis/gluconeogenesis, (C) fatty acid degradation, (D) citrate cycle, and (E) cardiac muscle contraction genes based on KEGG pathways (n=6). Dendrograms visualize hierarchical Z-score clustering. Control-D, Control / delivery; Control-NP, Control / nulliparous; Control-PP, Control / postpartum; iRhot1/2-KO-D, iRhot1/2-KO / delivery; iRhot1/2-KO-NP, iRhot1/2-KO / nulliparous; iRhot1/2-KO-PP, iRhot1/2-KO / postpartum.

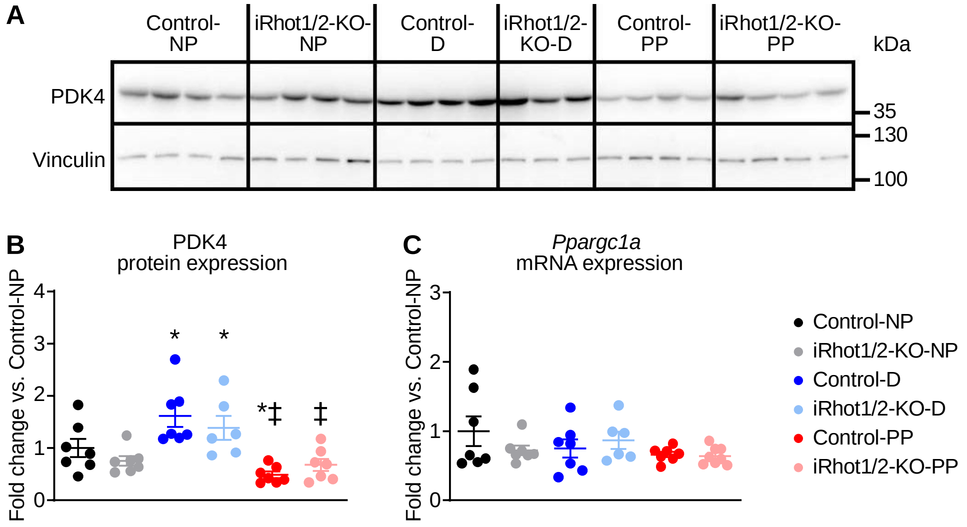

Figure S5. *Increased PDK4 protein expression at delivery independent of Rhot1/2 expression.* (A/B) Representative immunoblots of left ventricular homogenates and densitometric quantification of PDK4 protein expression normalized to vinculin and presented as fold change vs. Control-NP (n=6-7; #). (C) *Peroxisome proliferator activated receptor gamma coactivator 1 alpha* (*Ppargc1a,* PGC-1α) mRNA expression in left ventricular tissue normalized to *Rps16* and presented as fold change vs. Control-NP (n=6-8). Data are reported as mean values ± SEM. * $p<0.05$ vs. nulliparous (NP) same genotype, ‡ $p<0.05$ vs. delivery (D) same genotype. Two-way ANOVA was performed to analyze differences by time point and genotype, followed by Holm-Šídák post hoc analysis (# $p<0.05$ for time point). Control-D, Control / delivery; Control-NP, Control / nulliparous; Control-PP, Control / postpartum; iRhot1/2-KO-D, iRhot1/2-KO / delivery; iRhot1/2-KO-NP, iRhot1/2-KO / nulliparous; iRhot1/2-KO-PP, iRhot1/2-KO / postpartum.

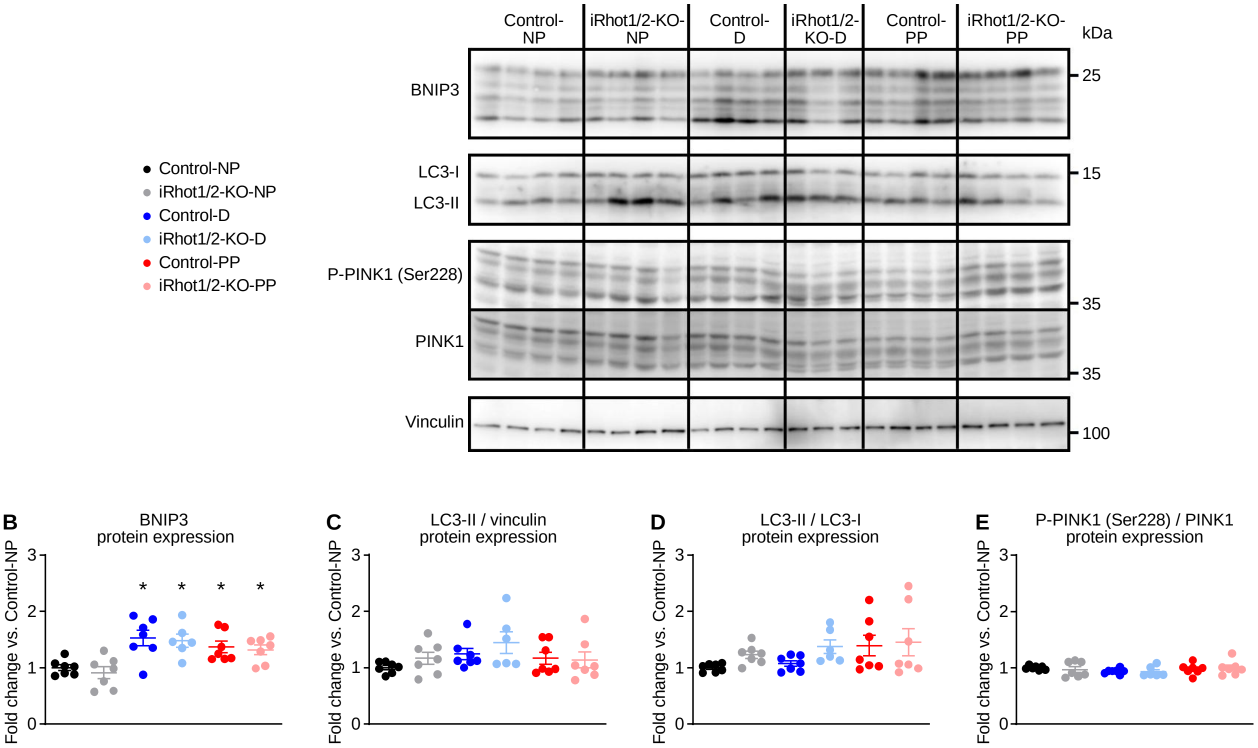

Figure S6. *Increased mitophagy, but not autophagy, at delivery and postpartum independent of Rhot1/2 expression.*
(A) Representative immunoblots of left ventricular homogenates and densitometric quantification of (B) BNIP3 normalized to vinculin (#), (C) LC3-II normalized to vinculin, (D) LC3-II normalized to LC3-I, and (E) P-PINK1 (Ser228) / PINK1 protein expression and presented as fold change vs. Control-NP (n=6-7). Data are reported as mean values ± SEM. * $p<0.05$ vs. nulliparous (NP) same genotype. Two-way ANOVA was performed to analyze differences by time point and genotype, followed by Holm-Šídák post hoc analysis (# $p<0.05$ for time point). Control-D, Control / delivery; Control-NP, Control / nulliparous; Control-PP, Control / postpartum; iRhot1/2-KO-D, iRhot1/2-KO / delivery; iRhot1/2-KO-NP, iRhot1/2-KO / nulliparous; iRhot1/2-KO-PP, iRhot1/2-KO / postpartum.

Supplementary Table S1. *Contractile function as assessed by transthoracic echocardiography.*

Group (n)	Heart Rate [bpm]	Ejection fraction [%]	LVEDA [mm^2] #	LVESA [mm^2] #	Fractional area change [%]
Control-NP (7)	536.5 ± 15.4	55.0 ± 4.1	20.6 ± 1.6	13.1 ± 1.5	36.9 ± 3.5
iRhot1/2-KO-NP (7)	571.6 ± 24.0	61.9 ± 4.5	20.0 ± 0.8	11.6 ± 1.0	42.6 ± 3.4
Control-D (7)	551.1 ± 19.0	56.9 ± 3.9	24.7 ± 1.3	15.6 ± 1.4	37.5 ± 2.9
iRhot1/2-KO-D (6)	529.4 ± 11.4	50.7 ± 2.8	25.4 ± 1.2 *	16.9 ± 0.8 *	33.2 ± 2.2
Control-PP (7)	501.3 ± 19.7	57.3 ± 3.1	26.1 ± 1.2 *	15.7 ± 0.7	39.6 ± 2.4
iRhot1/2-KO-PP (8)	519.0 ± 23.7	55.9 ± 3.4	25.6 ± 1.3 *	16.0 ± 1.4 *	38.2 ± 2.4

Data are reported as mean values ± SEM. * $p<0.05$ vs. nulliparous (NP) same genotype. Two-way ANOVA was performed to analyze differences by time point and genotype, followed by Holm-Šídák post hoc analysis (# $p<0.05$ for time point). Bpm, beats per minute; Control-D, Control / delivery; Control-NP, Control / nulliparous; Control-PP, Control / postpartum; iRhot1/2-KO-D, iRhot1/2-KO / delivery; iRhot1/2-KO-NP, iRhot1/2-KO / nulliparous; iRhot1/2-KO-PP, iRhot1/2-KO / postpartum; LVEDA, left ventricular end-diastolic area; LVESA, left ventricular end-systolic area.

Supplementary Table S2. *Morphometrics of mice.*

Group (n)	Body weight [g] #, &	Heart weight [mg] #, &	Tibia length [mm]	Heart weight / tibia length [mg/mm] #
Control-NP (7)	28.7 ± 0.8	111.1 ± 2.8	18.4 ± 0.1	6.06 ± 0.15
iRhot1/2-KO-NP (7)	29.4 ± 1.1	125.3 ± 3.5	18.9 ± 0.2	6.63 ± 0.18
Control-D (7)	34.0 ± 0.4 *	153.0 ± 2.5 *	18.3 ± 0.3	8.38 ± 0.23 *
iRhot1/2-KO-D (6)	35.1 ± 0.9 *	158.8 ± 5.9 *	18.9 ± 0.9	8.48 ± 0.43 *
Control-PP (7)	40.7 ± 1.4 *‡	182.4 ± 5.1 *‡	19.0 ± 0.3	9.65 ± 0.40 *‡
iRhot1/2-KO-PP (8)	36.1 ± 1.2 *†	166.5 ± 8.3 *	19.0 ± 0.3	8.78 ± 0.47 *

Data are reported as mean values ± SEM. * $p<0.05$ vs. nulliparous (NP) same genotype, † $p<0.05$ vs. Control same time point, ‡ $p<0.05$ vs. delivery (D) same genotype. Two-way ANOVA was performed to analyze differences by time point and genotype, followed by Holm-Šídák post hoc analysis (# $p<0.05$ for time point and & $p<0.05$ for the interaction between time point and genotype). Control-D, Control / delivery; Control-NP, Control / nulliparous; Control-PP, Control / postpartum; iRhot1/2-KO-D, iRhot1/2-KO / delivery; iRhot1/2-KO-NP, iRhot1/2-KO / nulliparous; iRhot1/2-KO-PP, iRhot1/2-KO / postpartum.

Supplementary Table S3. *Supplemental dataset (RNA sequencing).*
See separate Excel file.

Supplementary Table S4. *Top canonical pathways identified by Ingenuity Pathway Analysis (IPA).*

Canonical Pathway	p-value	Overlap, % (Ratio)
**VII – iRhot1/2-KO-NP vs. Control-NP**		
ILK signaling	8.15E-05	4.0% (8/201)
IL-8 signaling	1.11E-04	3.8% (8/210)
Response of EIF2AK1 (HRI) to heme deficiency	1.39E-04	20.0% (3/15)
Glioma invasiveness signaling	1.52E-04	6.8% (5/73)
Glioblastoma multiforme signaling	1.92E-04	4.1% (7/171)
**VIII – iRhot1/2-KO-D vs. Control-D**		
Mitochondrial dysfunction	4.60E-12	13.7% (47/344)
Estrogen receptor signaling	1.83E-11	12.5% (51/409)
Electron transport, ATP synthesis, and heat production by uncoupling proteins	1.48E-09	18.8% (24/128)
Oxidative phosphorylation	2.85E-09	19.6% (22/112)
Deubiquitination	1.72E-08	12.9% (34/263)
**IX – iRhot1/2-KO-PP vs. Control-PP**		
EIF2 signaling	8.29E-26	23.5% (54/230)
Eukaryotic translation initiation	2.17E-25	32.8% (40/122)
Selenoamino acid metabolism	3.23E-19	29.9% (32/107)
Eukaryotic translation termination	5.33E-19	31.9% (30/94)
Nonsense-mediated decay (NMD)	6.66E-19	28.2% (33/117)

The table shows the top five canonical pathways for the indicated contrasts (cutoff: FDR<0.1; n=6). Control-D, Control / delivery; Control-NP, Control / nulliparous; Control-PP, Control / postpartum; iRhot1/2-KO-D, iRhot1/2-KO / delivery; iRhot1/2-KO-NP, iRhot1/2-KO / nulliparous; iRhot1/2-KO-PP, iRhot1/2-KO / postpartum.

Supplementary Table S5. *Antibodies used for immunoblotting.*

Antigen	Company	Catalog number	RRID
4-HNE	Abcam, Cambridge, A, USA	46545	AB_722490
BNIP3	Cell Signaling, Danvers, MA, USA	3769	AB_2259284
DRP1	Novus Biologicals, Centennial, CO, USA	h00010059-m01	AB_538285
LC3	Sigma-Aldrich, St. Louis, MO, USA	L7543	AB_796155
MFN1	Abcam, Cambridge, MA, USA	ab57602	AB_2142624
MFN2	Sigma-Aldrich, St. Louis, MO, USA	M6319	AB_477221
MnSOD	BD Biosciences, San Jose CA, USA	611580	AB_399026
OPA1	BD Biosciences, San Jose CA, USA	612607	AB_399889
PDK4	Abcam, Cambridge, A, USA	ab214938	AB_2864318
PINK1	Proteintech, Rosemont, IL, USA	23274-1-AP	AB_2879244
P-PINK1 (Ser228)	Cell Signaling, Danvers, MA, USA	46421	n/a
RHOT1	Atlas Antibodies, Bromma, Sweden	HPA010687	AB_1079813
RHOT2	NeuroMab, Davis, CA, USA	75-365	AB_2315894
VDAC	Thermo Fisher Scientific, Waltham, MA, USA	PA1-954A	AB_2304154
Vinculin	Santa Cruz Biotechnology, Santa Cruz, CA, USA	7649	AB_2288413
Rabbit anti-goat HRP-linked	Jackson ImmunoResearch, West Grove, PA, USA	305-035-045	AB_2339403
Sheep anti-mouse HRP-linked	GE HealthCare, Chicago, IL, USA	NXA931V	AB_772209
Donkey anti-rabbit HRP-linked	GE HealthCare, Chicago, IL, USA	NA934V	AB_772206

Supplementary Table S6. *Primers used for RT-PCR analysis.*

**Gene Name *(Gene Symbol)*<br>Sequence of forward and<br>reverse primers (5' → 3')**
Actin. alpha 1. skeletal muscle *(Acta1)*<br>GCCAGAGTCAGAGCAGCAGAAACTA<br>CAGAGCCGTTGTCACACACAAGA
Hypoxanthine phosphoribosyltransferase 1 *(Hprt1)*<br>GGACCTCTCGAAGTGTTGGATAC<br>GCTCATCTTAGGCTTTGTATTTGGCT
Natriuretic peptide type A *(Nppa)*<br>ATTGACAGGATTGGAGCCCAGAGT<br>TGACACACCACAAGGGCTTAGGAT
Natriuretic peptide type B *(Nppb)*<br>CTCAAGCTGCTTTGGGCACAAGAT<br>AGCCAGGAGGTCTTCCTACAACAA
Peroxisome proliferator activated receptor, gamma, coactivator 1 alpha *(Ppargc1a)*<br>GTAAATCTGCGGGATGATGG<br>AGCAGGGTCAAAATCGTCTG
Ras homolog family member T1 *(Rhot1)*<br>ATTGGAGTGAAAGGCTGTGG<br>AATAGGACTTATGATCATCACGAATTT
Ras homolog family member T2 *(Rhot2)*<br>GGAGAAGGTGCCCACTCATA<br>TCCACTTGGTTCGGATCTTC
Ribosomal protein S16 *(Rps16)*<br>TGCTGGTGTGGATATTCGGG<br>CCTTGAGATGGGCTTATCGG
Vascular endothelial growth factor A *(Vegfa)*<br>CAAGATCCGCAGACGTGTAA<br>TTAATCGGTCTTTCCGGTGA
